# Evaluation of Melanoma (SK-MEL-2) Cell Growth between Three-Dimensional (3D) and Two-Dimensional (2D) Cell Cultures with Fourier Transform Infrared (FTIR) Microspectroscopy

**DOI:** 10.3390/ijms21114141

**Published:** 2020-06-10

**Authors:** Tarapong Srisongkram, Natthida Weerapreeyakul, Kanjana Thumanu

**Affiliations:** 1 Research and Development in Pharmaceuticals Program, Graduate School, Faculty of Pharmaceutical Sciences, Khon Kaen University, Khon Kaen 40002, Thailand; tarapong.sri@gmail.com; 2 Division of Pharmaceutical Chemistry, Faculty of Pharmaceutical Sciences, Khon Kaen University, Khon Kaen 40002, Thailand; 3 Human High Performance and Health Promotion Research Institute, Khon Kaen University, Khon Kaen 40002, Thailand; 4 Synchrotron Light Research Institute (Public Organization), Nakhon Ratchasima 30000, Thailand; kthumanu@gmail.com

**Keywords:** three-dimensional (3D) cell culture, Fourier transform infrared microspectroscopy (FTIR), melanoma, multivariate analysis, spheroid cell culture, colony formed cells

## Abstract

Fourier transform infrared (FTIR) microspectroscopy was used to evaluate the growth of human melanoma cells (SK-MEL-2) in two-dimensional (2D) versus three-dimensional (3D) spheroid culture systems. FTIR microspectroscopy, coupled with multivariate analysis, could be used to monitor the variability of spheroid morphologies prepared from different cell densities. The characteristic shift in absorbance bands of the 2D cells were different from the spectra of cells from 3D spheroids. FTIR microspectroscopy can also be used to monitor cell death similar to fluorescence cell staining in 3D spheroids. A change in the secondary structure of protein was observed in cells from the 3D spheroid versus the 2D culture system. FTIR microspectroscopy can detect specific alterations in the biological components inside the spheroid, which cannot be detected using fluorescence cell death staining. In the cells from 3D spheroids, the respective lipid, DNA, and RNA region content represent specific markers directly proportional to the spheroid size and central area of necrotic cell death, which can be confirmed using unsupervised PCA and hierarchical cluster analysis. FTIR microspectroscopy could be used as an alternative tool for spheroid cell culture discrimination, and validation of the usual biochemical technique.

## 1. Introduction

Malignant melanoma is a leading cause of death worldwide, with over 10% mortality among cases each year [1]. Multiple gene mutations in melanoma cells leads to an increase in chemotherapy resistance and thus the greater mortality [2]. Such characteristics have led researchers to develop novel and targeted therapies for suppressing melanoma progression [2,3]. Disease modeling in melanoma studies is usually based on an adherent (two-dimensional (2D)) cell culture, however, such cultures do not mimic the characteristics of a clinical tumor well [4]. Three-dimensional (3D) cell cultures provide better outcomes vis-à-vis drug development and disease modeling [5]. 3D cell culture systems are a non-animal alternative and yield more clinically relevant information on primary cancer tissue and therapeutic outcomes than do 2D cell culture systems [5]. Based on these advantages, the current stage assessments of in vitro melanoma now employ 3D cell culture systems [3]. 

Even though 3D cell cultures have several advantages, inconsistencies in spheroid formation (i.e., volume and shape variability) affect dermal drug or cosmetic product responses [6,7]. Biotechnology tools are used to determine the source(s) of size and shape inconsistencies; namely, quantitative polymerase chain reaction (qPCR) [8], immunohistochemistry evaluation [9], and immunofluorescence staining [10]. These techniques, however, require several costly steps for sample preparation and manipulation. An alternative technique was tested for monitoring the functional consistency and quality of each batch of spheroids. Vibrational spectroscopy (i.e., FTIR or Raman microspectroscopy) can be used to assess differences in spheroids at the molecular level and compared to classic techniques as it is faster, less expensive, and requires less sample preparation [11]. FTIR microspectroscopy is highly correlated with biological testing [8], is label free [12], and can clinically discriminate the primary tumor [13]. It is interesting to test whether FTIR microspectroscopy can be used for monitoring of spheroid variability.

In the current study, FTIR microspectroscopy—validated by multivariate analysis—was used to track the differences between 2D and 3D spheroid culture systems of SK-MEL-2 cells and to discriminate between differences in the SK-MEL-2 spheroids. The SK-MEL-2 cell line was selected because of its being clinically relevant to melanoma cells. In the current study, FTIR data represent the characteristic differences between the 2D cell culture and 3D spheroids and their respective ability to distinguish variations in the 3D spheroids. Immunofluorescence staining is, moreover, a well-known approach for evaluating 3D spheroid culture systems.

## 2. Results

### 2.1. Discrimination of Morphology of 2D and 3D Cells’ Culture 

**Figure 1:**
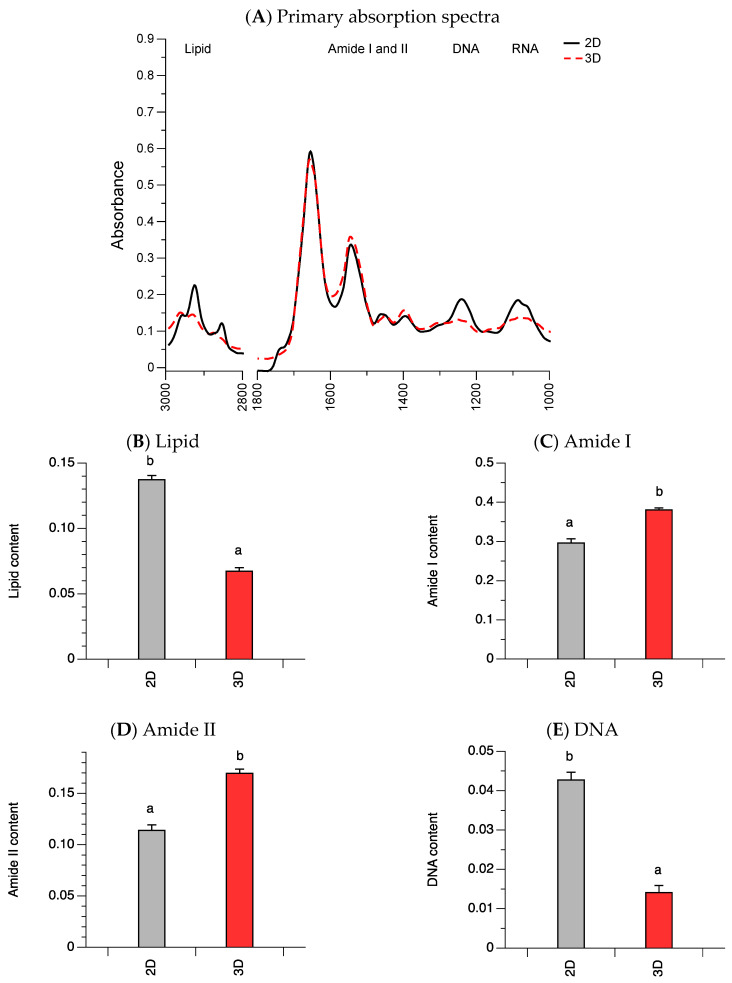

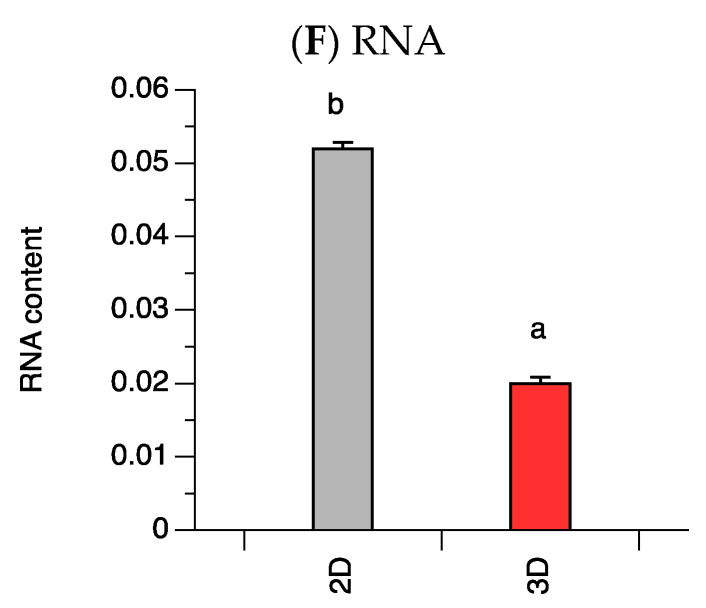
The primary absorption spectra and integration ratios of the five spectral regions (2813–2992 cm^−1^, 1600–1700 cm^−1^, 1500–1600 cm^−1^, 1180–1280 cm^−1^, and 1040–1140 cm^−1^). (**A**) Average absorption spectra of the cell culture systems: two-dimensional (2D) (5000 cells/per replicate) (black; 76 spectra) and three-dimensional (3D) spheroids (5000 cells/per spheroid) (red; 73 spectra). (**B**–**F**) Integration ratios for each spectral region, including the lipid, amide I, amide II, DNA, and RNA (*n* = 3). Each different letter represents a statistical difference between groups (*p* < 0.05).

**Figure 2:**
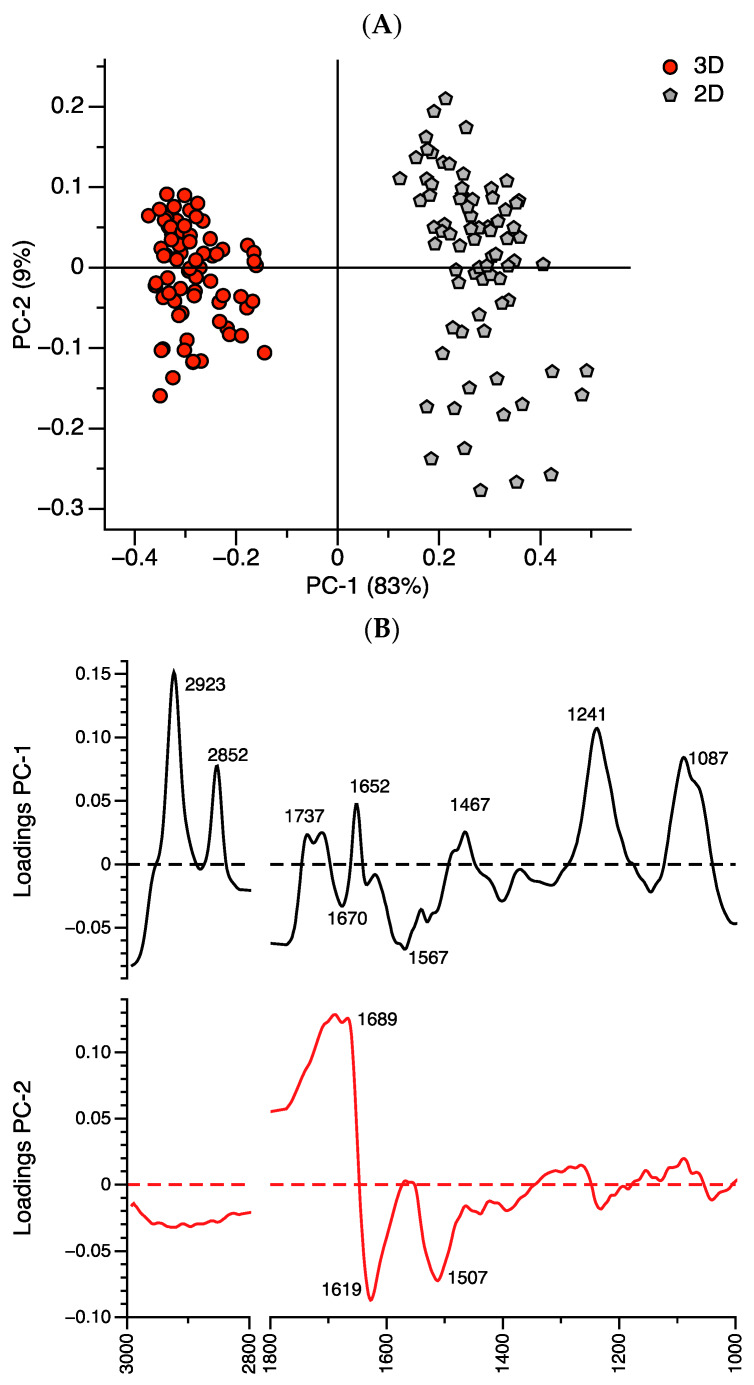

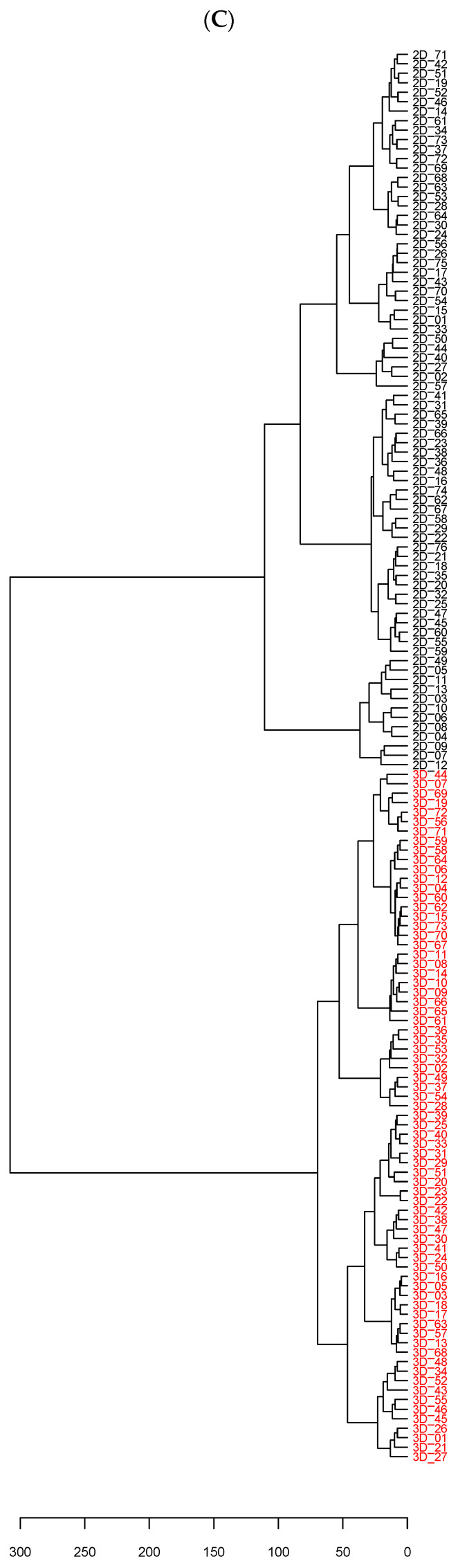
Multivariate analysis of primary absorption spectra between 2D cells (5000 cells/per replicate) (black; 76 spectra) and 3D spheroid cells (5000 cells/per spheroid) (red; 73 spectra). (**A**) Principal component analysis (PCA) between 2D cells and 3D spheroid cells. (**B**) Loading plot obtained from the PCA discrimination between 2D cells and 3D spheroid cells. (**C**) Cluster analysis based on Ward’s algorithm between 2D cells (black color) and 3D spheroid cells (red color).

Figure 1A shows the average absorbance spectra of 2D cell cultures versus 3D spheroids (5000 cells/per replicate) grown at 5 days. The initial cell seeding at 5000 cells per spheroid was used as this seeding rate was previously reported to create spheroids for various drug testing assays [14,15,16]. The absorbance spectra of FTIR can be used to represent the amounts of cellular biocomponents [17]. The absorbance spectra can be divided into five regions based on the biocomponents, including the lipid (2813–2992 cm^−1^), protein (amide I; 1600–1700 cm^−1^ and amide II; 1480–1600 cm^−1^), DNA (1180–1280 cm^−1^), and RNA (1040–1140 cm^−1^) regions (Figure 1A). The semiquantitative biocomponents inside the cells were the ratio of the specific band area integrals of each biocomponent and the overall cell biocomponents. The respective area integrals of each biocomponent (the lipid, Amide I, Amide II, DNA, and RNA region) were calculated, then corrected according to the baseline [18]. The overall cell biocomponents were the sum of the area integrals—the spectral ranges 2992–2813 cm^−1^ and 1772–937 cm^−1^ [18]. Figure 1B,F show the 5 integrated ratios of each biocomponent versus the overall cell biocomponents of the FTIR spectra between the 2D cells and 3D spheroid cells. The content of the respective lipid, DNA, and RNA region of the 2D cells was significantly greater than that of the 3D spheroid cells (Figure 1B–F). After transforming a 2D to a 3D spheroid culture system of breast cancer [19] and hepatocellular carcinoma cells [20], a significant decrease of the respective lipid, DNA, and RNA concentration was observed. In order to better understand the differentiation of 2D and 3D spheroid culture systems of melanoma cells, we analyzed the FTIR spectra using multivariate analysis (Figure 2A). 

Unsupervised principal component analysis (PCA) was used to identify clustering in the datasets and to prevent a classification bias. The PCA of the spectra of the 2D cells and 3D spheroid cells revealed a clear separation into two first Principal Components (PC-1 and PC-2, Figure 2A). The cluster of 3D spheroid cells was well-separated from the clusters of the 2D cells vis-à-vis PC1 (83%) and PC2 (9%). The FTIR spectra of the 3D spheroid cells were clustered into a PC-1 negative region while the FTIR spectra of the 2D cells were clustered into a PC-1 positive region (Figure 2A). The major variables (wavenumber) contributing to the separation of the 2D cells and 3D spheroid cells were indicated by the loading plot (Figure 2B). The heavy loading for the PC-1 positive (2D cells) comprised: (1) 2852 and 2923 cm^−1^ (the symmetric and asymmetric stretching vibration (*v*_s_ and *v*_as_) region of the CH_2_- of the alkyl chain of the lipid) [21], (2) 1652 cm^−1^ (the C=O, C-N, N-H of the α-helix in amide I) and 1737 cm^−1^ (the stretching vibration (*v*) of the C=O of the lipid band) [22], (3) 1467 cm^−1^ (the CH_3_ bending of the lipid or protein regions) [11], and (4) 1087 and 1241 cm^−1^ (the asymmetric (*v*_as_) and symmetric (*v*_s_) stretching vibration of the PO_2_^−^ of the phosphodiester backbone of the DNA and RNA) [21]. By comparison, the heavy loading of the PC-1 negative (i.e., 3D spheroid cells) was 1670 and 1567 cm^−1^ (the CN-H and C-N β-turns in amide I and II) [23]. These data suggest that 2D cells have more lipid, α-helix, DNA, and RNA than the 3D spheroid cells but fewer β-turns than the 3D spheroid cells. Thus, the lipid, protein secondary structure (especially α-helix), DNA, and RNA are most strongly associated with discrimination of the melanoma 2D cells from 3D spheroid cells.

To confirm the multivariate analysis results, we performed an unsupervised hierarchical cluster analysis of the 2D cells and 3D spheroid cells (5000 cells/per spheroid), using Ward’s algorithm (Figure 2C). The results showed that the spectra from the 2D cells and 3D spheroids were different. From a total of 149 cell spectra, 76 spectra of the 2D cells (spectra numbers 1 to 76) (76/76; 100%) were clustered into the same group, while 73 spectra of the 3D spheroids (spectra numbers 77 to 149) (73/73; 100%) were clustered together (Figure 2C). Cluster analysis confirmed that the major discriminators (wavenumbers) between the 2D cells from 3D spheroids were the specific bands at 2852 and 2923 cm^−1^ of the alkyl chain of the lipid group, 1652 cm^−1^ of the α-helix in amide I, 1241 cm^−1^, and 1087 cm^−1^ of the phosphodiester backbone of the DNA and RNA (Figure 2B).

### 2.2. 3D Cells’ Spheroid Variability

**Figure 3:**
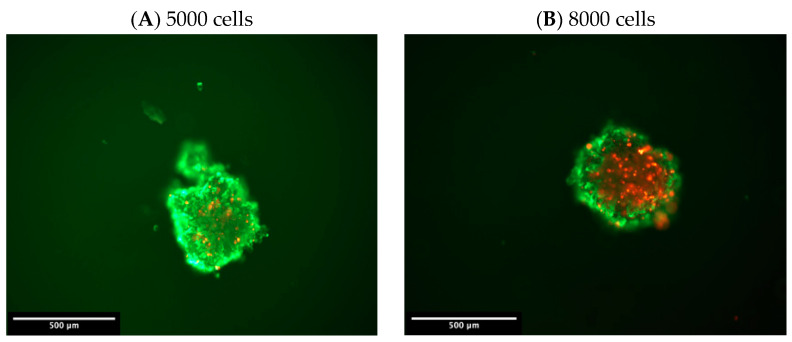

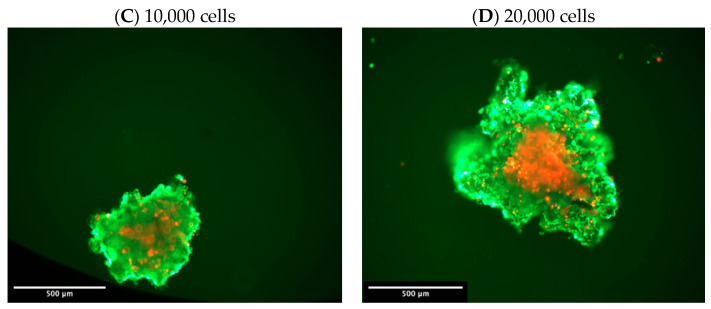
Morphology of the spheroid at various cell densities initially seeded at (**A**) 5000 cells, (**B**) 8000 cells, (**C**) 10,000 cells, and (**D**) 20,000 cells. Images were captured at a scale bar of 500 µm and 10X magnification. Red and green represent propidium iodide and annexin V staining, which in turn indicates the area of necrotic and apoptotic cell death, respectively.

Figure 3A,D illustrate the morphological differences among the four groups of melanoma spheroids after culturing for 5 days. The spheroids were created by using different initial cell numbers for seeding, as per Wang et al. [14] and Shannan et al. [15]. Cells were initially seeded at 5000, 8000, 10,000, and 20,000 cells per spheroid. The volume of the spheroid was significantly increased from 40.3 ± 5.4 mm^3^ to 97.7 ± 2.0 mm^3^ when the initial cell numbers were increased from 5000 to 20,000 cells (Table 1). The quality of the spheroids was determined based on the measurement of living cells versus dead cells by fluorescence staining using annexin V (green) (for apoptosis induction) and propidium iodide (red) (for necrosis induction). The necrotic area was increased from 0.7 to 1.4 (× 10^7^) corrected total cellular fluorescence (CTCF) when the cell number was increased from 5000 to 10,000 cell/spheroid and increased to 4.0 (× 10^7^) CTCF in 20,000 cells/spheroid (Table 1). It should be noted that necrotic cells were increased when the cell number and size of the spheroid were increased. Apoptotic cell death was increased nominally from 10,000 to 20,000 cells/spheroid (Table 1). The results suggest that increasing cell number up to 20,000 cells per spheroid and size (97.7 mm^3^) resulted in relatively more necrotic cell death than apoptotic cell death (Table 1 and Figure 3). The cell number—up to 20,000 cells—affected cell death and thus affected the final quality of the melanoma spheroids.

**Figure 4:**
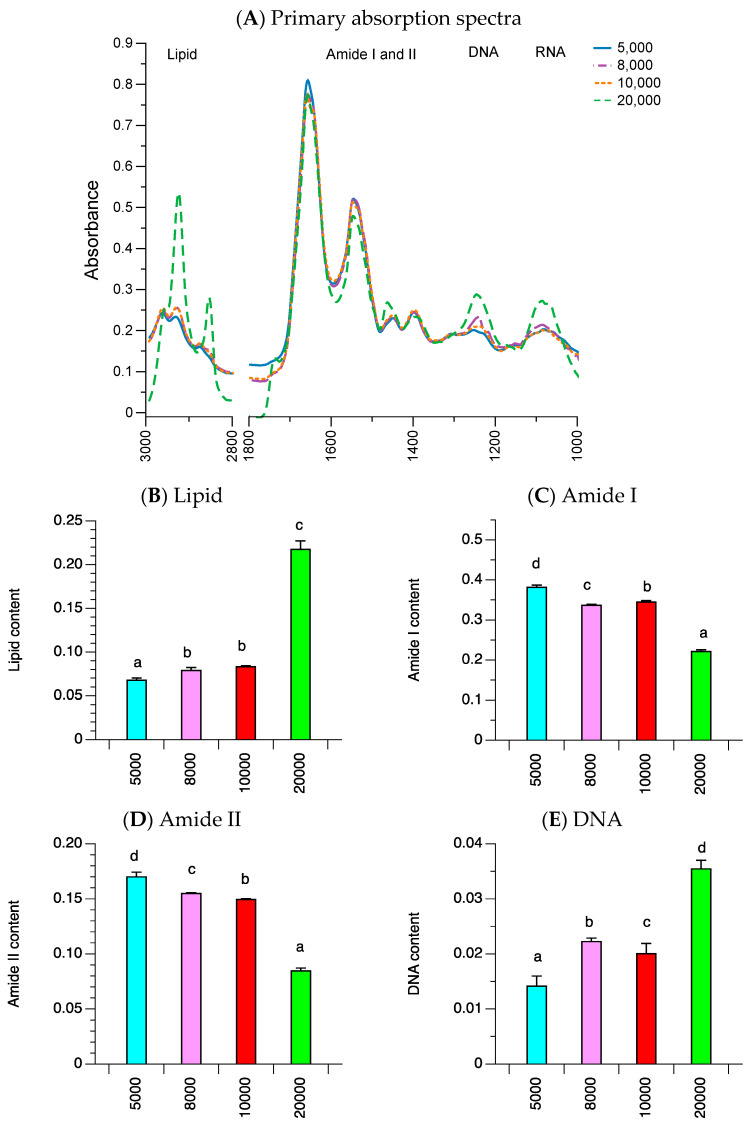

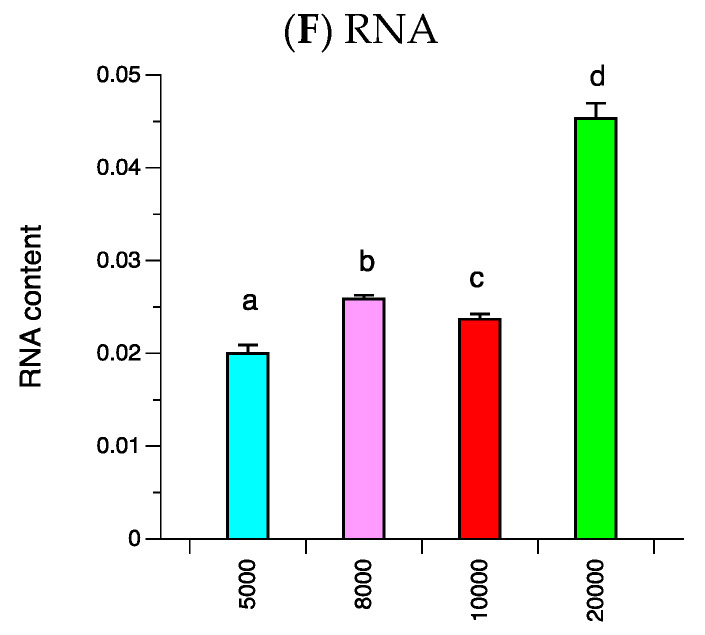
Fourier transform infrared (FTIR) primary spectra and integration area of cells from various spheroids. (**A**) Spheroid cells were initially seeded at various cell densities (5000 cells (solid blue line; 73 spectra), 8000 cells (purple dot-dashed line; 61 spectra), 10,000 cells (red dotted line; 60 spectra), and 20,000 cells (green dashed line; 62 spectra)). The FTIR primary spectra was divided into five spectral regions: the lipid (2813–2992 cm^−1^), amide I (1600–1700 cm^−1^), amide II (1480–1600 cm^−1^), DNA (1180–1280), and RNA (1040–1140 cm^−1^) region. The average, respective absorption spectra of each spectral region was integrated and corrected according to the total integration area, as shown in (**B**–**F**) for the lipid, amide I, amide II, DNA, and RNA region (n = 3). Different letters indicate a statistical difference between groups (*p*-value < 0.05).

The FTIR results in Figure 4A show the average absorbance spectra for spheroids at different cell seeding densities, illustrating the five biocomponent regions (lipid, protein (amide I and II), DNA, and RNA region). The variation in cells from spheroids were determined from the standard deviation (SD) of the spectra under each condition. The positive SDs for the lipid region (2813–2992 cm^−1^), protein region, and nucleic acids region (1000–1700 cm^−1^) for all the spheroid groups ranged between 0.0132 and 0.0548, indicating good uniformity of the spheroid samples. The area integral ratios for each biocomponent versus the overall cell biocomponents of the spheroid spectra are shown in Figure 4B–F. The respective lipid, DNA, and RNA region content of the spheroids increased when the cell seeding density of the spheroids increased from 5000 to 20,000 cells (Figure 4B,E,F). The respective amide I and amide II region content decreased when the cell seeding number increased from 5000 to 20,000 cells/spheroid (Figure 4C,D). The results show a moderate correlation between the spheroid volume and the respective lipid, DNA, and RNA region content (respective Pearson’s correlation coefficient (*r*) = +0.697, +0.763, and +0.619 (*p* < 0.05), Supplementary Table S1). There was a strong correlation between necrotic cell death and the respective lipid, DNA, and RNA region content (*r =* +0.987, +0.928, and +0.912 (*p* < 0.001), respectively). There was a strong inverse correlation between spheroid volume and the amide I and amide II region content (*r* = −0.800 and −0.798 (*p* = 0.002), respectively), and between necrotic cell death and the amide I and amide II region content (*r* = −0.997 and −0.992 (*p* < 0.001), respectively). Our study suggests that increasing the spheroid volume can increase necrotic cell death as well as the respective lipid, DNA, and RNA region content, while reducing the protein region content.

**Figure 5:**
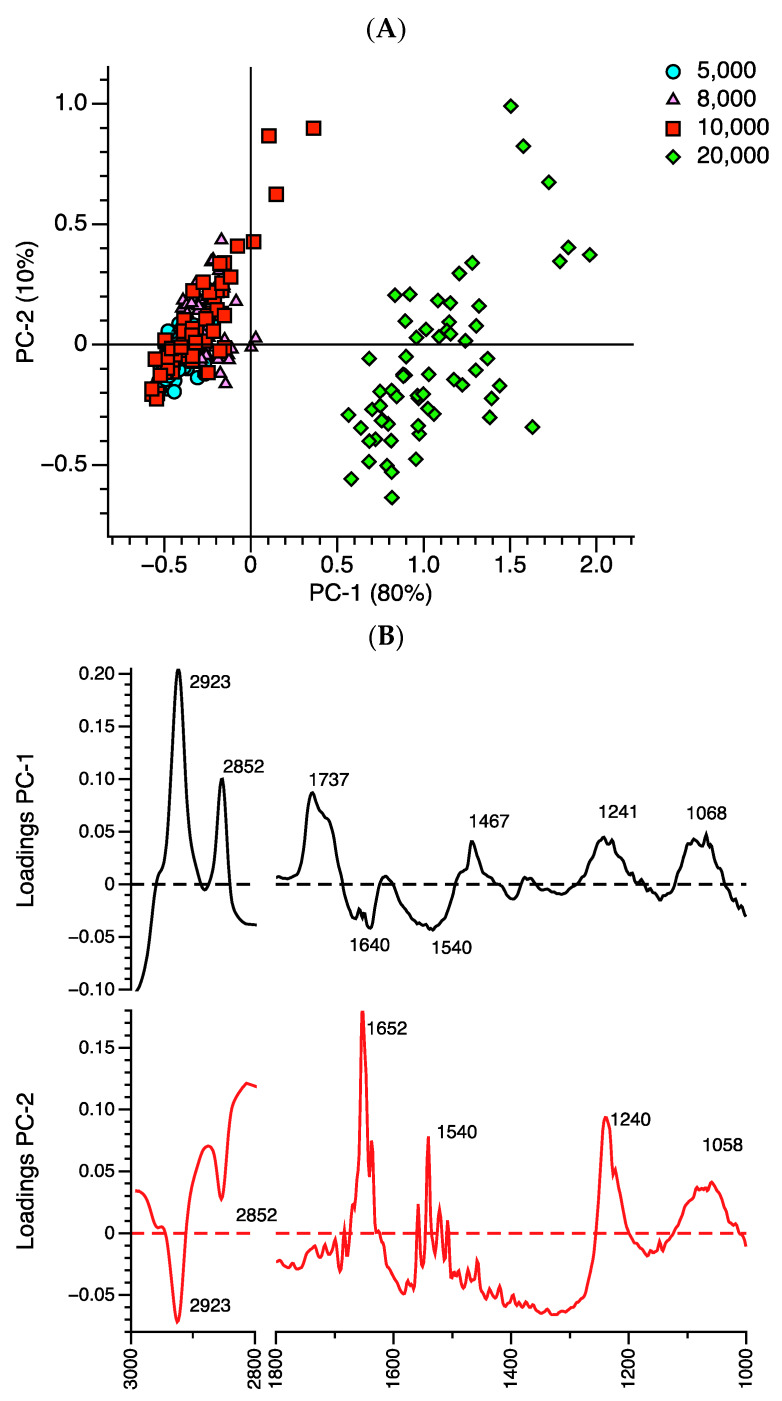

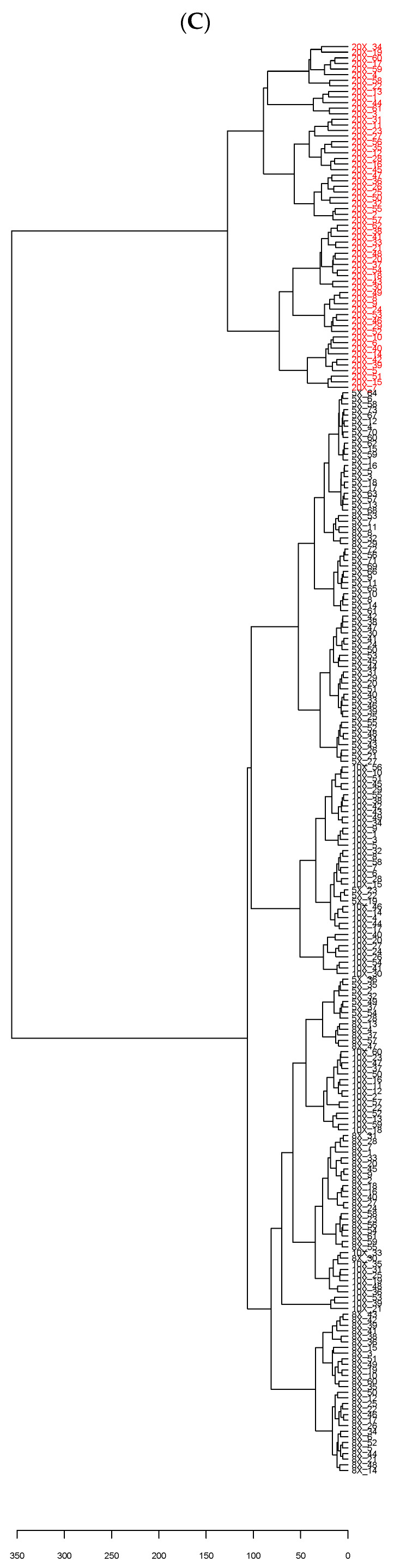
Unsupervised multivariate analysis of primary absorption spectra between the groups of 5000 (●; 73 spectra), 8000 (▲; 61 spectra), 10,000 (■; 60 spectra), and 20,000 (◆; 62 spectra) spheroid cells. (**A**) Principal component analysis (PCA) and (**B**) loading plots were obtained from the PCA discrimination of the spheroid cell groups. (**C**) Cluster analysis between groups of 3D spheroid cells was performed using Ward’s algorithm. Red color indicates 20,000 spheroid cells while black color is a combination of 5000 (5×), 8000 (8×), and 10,000 (10×) spheroid cells.

The unsupervised PCA of primary spectra among the groups of spheroid cells are shown in Figure 5A. The PCA revealed a clear separation into two clusters: first principal components, which were primarily separated by PC-1 (80%) and less so by PC-2 (10%) (Figure 5A). The 20,000 spheroid cells were clearly separated into PC-1 positive while the other spheroid cell groups (5000–10,000 spheroid cells) were clustered into PC-1 negative. Figure 5B represents the major variables (wavenumber) for the classification between the spheroid cell groups. The heavy loading for the PC-1 positive comprised wave number 2852 and 2923 cm^−1^ (assigned as *v*_s_ and *v*_as_ CH_2_– of the alkyl chain of the lipid) [21], 1737 cm^−1^ (*v* (C=O) of the lipid) [22], 1467 cm^−1^ (CH_3_ of the lipid/protein [11], 1241 cm^−1^ (*v*_as_ and *v*_s_ PO^2–^ of the phosphodiester backbone of the DNA) [21], and 1068 cm^−1^ (*v*_s_ (C-O) of RNA ribose) [11]. The heavy loading for PC-1 negative was 1640 cm^−1^ (C=O, C-N, N-H of the random coil in amide I) [23], and 1540 cm^−1^ (assigned as CN-H, N-H of the β-sheet in amide II) [11]. In addition, the 2852 cm^−1^ of CH_2_ of the lipid, 1652 cm^−1^ of the α-helix, 1540 cm^−1^ of the β-sheet, 1240 cm^−1^ of the DNA, and 1058 cm^−1^ of the RNA contributed to the PC-2 positive (Figure 5B). The 20,000 spheroid cells were primarily separated from the 5000 to 10,000 spheroid cells by the higher alkyl chain of lipid (CH_2_; 1737, 2852 and 2923 cm^−1^ and CH_3_; 1467 cm^−1^), and the DNA (1241 cm^−1^) and RNA (1068 cm^−1^) content. These findings agree with the results of area integrals of the spheroid cells, which indicated that 20,000 spheroid cells have a higher lipid, DNA, and RNA content, but lower amide I and amide II content (Figure 4B–F).

The unsupervised hierarchical cluster analysis of the 3D spheroid cells using Ward’s algorithm (Figure 5C) was performed to confirm the band contributors of the PCA result. The cluster analysis shows that the spectra of the 256 spheroid cells can be classified into two groups. Sixty-two spheroid cells (numbers 194 to 256) of the 20,000 spheroid cells (100%, 62/62) were clearly clustered into the same group. The 194 other spheroid cells (numbers 1 to 194; 5000, 8000, 10,000) (194/194) were clustered together (Figure 5C). No separate clustering of spheroid cells—from the seeding cell groups between 5000 to 10,000 spheroid cells—was observed, indicating the similarity of cell morphology and biochemical composition of the spheroids. Clear separate clusters of spheroid cells were found when there was increased seeding of cells (i.e., up to 20,000 spheroid cells), as found in the PCA analysis (Figure 5A). The cluster analysis confirmed that the major discriminators between spheroid cells were the specific bands at 2852, 2923 cm^−1^ of alkyl chain of lipid, 1640 cm^−1^ of random coil structure, 1068 cm^−1^ of RNA, and 1241 cm^−1^ of phosphodiester backbone of DNA (Figure 5B).

### 2.3. Principle Component Analyisis (PCA) between Melanoma 2D and All Shperoid Groups

**Figure 6:**
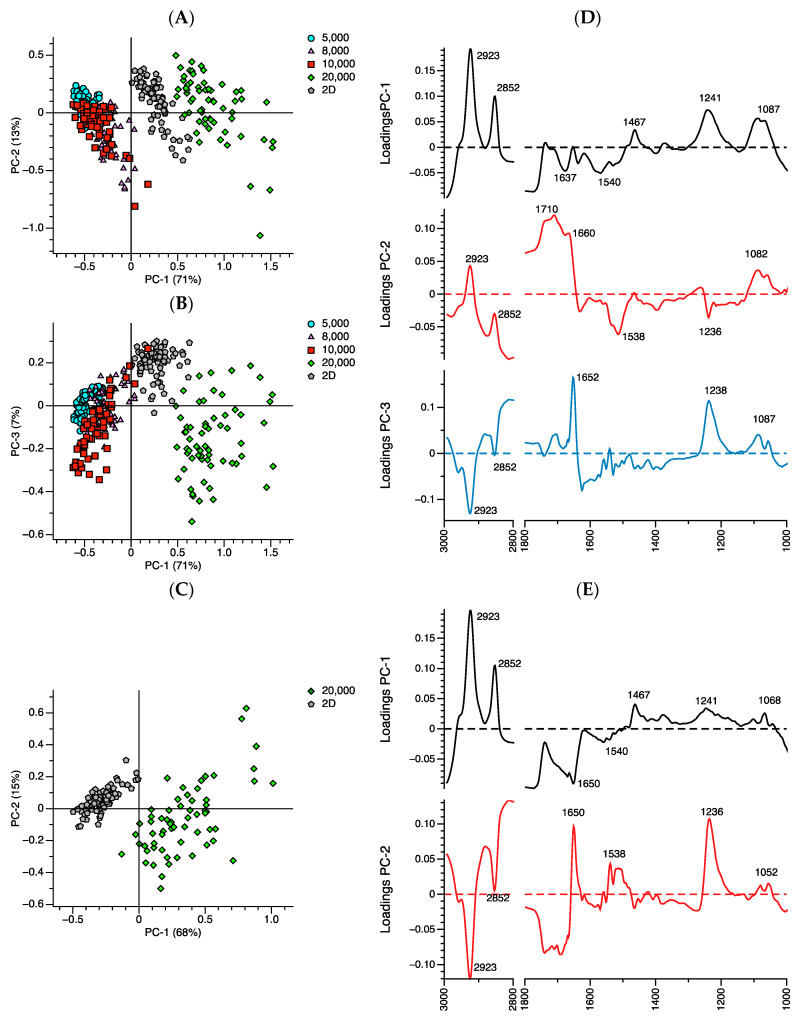
Unsupervised multivariate analysis of primary absorption spectra between the 2D (⬟), 5000 (●), 8000 (▲), 10,000 (■), and 20,000 (◆) groups of spheroid cells. (**A**) Principal component analysis (PCA) between PC-1 and PC-2, (**B**) principal component analysis (PCA) between PC-1 and PC-3, (**C**) principal component analysis of 2D and 20,000 spheroid cells. (**D**) Loading plots were obtained from the PCA discrimination between 2D and all spheroid cell groups and (**E**) between 2D and 20,000 spheroid cells.

To further investigate the discrimination between 2D cells and all 3D spheroid cells, the PCA analysis of the primary spectra between the 2D groups and groups of 3D spheroid cells was evaluated (Figure 6A–E). Figure 6A,B show that the clusters of 2D melanoma cells and 20,000 spheroid cells separated from the other groups of spheroid cells along PC-1 (71%), while the spectra of 2D cells were separated from the 20,000 spheroid cells along PC-3 (7%) (Figure 6B). When PCA analysis was performed between 2D and 20,000 spheroid cells (Figure 6C), the spectra of the 2D melanoma cells were significantly different from the clusters of 20,000 spheroid cells along PC-1 (68%).

**Figure 7:**
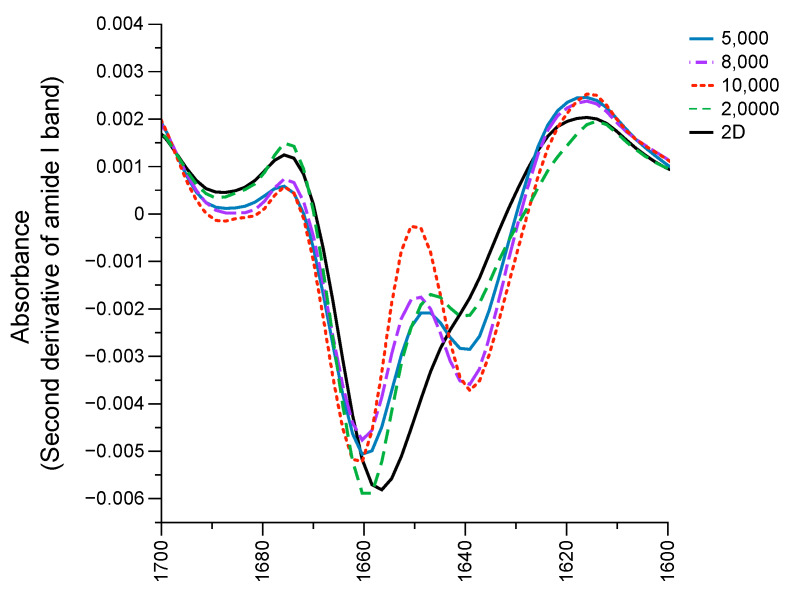
Second derivative of average spectra in the amide I region (between 1600 and 1700 cm^−1^) between 2D cells (solid black line), 5000 spheroid cells (solid blue line), 8000 spheroid cells (purple dot-dashed line), 10,000 spheroid cells (red dotted line), and 20,000 spheroid cells (green dashed line).

The loading plot of melanoma 2D and all the groups of spheroid cells (Figure 6D) demonstrated that the major biocomponents that contributed to the discrimination of 2D cells from 20,000 spheroid cells along PC3 (Figure 6B,D) comprised the amide I band from proteins assigned as an α-helix structure (1652 cm^−1^). In addition, the data of the PC-3 loading plot (Figure 6D) indicates that the α-helix structure (1652–1660 cm^−1^) of the 2D cells was higher than the spectra of the 20,000 spheroid cells. This result was confirmed by the second derivative of the amide I band (Figure 7), where the spectra between the 2D cells and the 3D spheroid cells were different at the peak position between 1630 and 1660 cm^−1^. 

The loading plot (Figure 6E) component that contributed to the discrimination of 20,000 spheroid cells from 2D cells (along PC-1) comprised the following regions: lipid (2852 and 2923 cm^−1^), α-helix (1650 cm^−1^), β-sheet (1538–1540 cm^−1^), and phosphodiester bond of DNA (1241 cm^−1^). The data confirmed that 2D cells had a higher α-helix structure than the 20,000 spheroid cells, whereas the 20,000 spheroid cells had a higher content of lipid and phosphodiester bond of DNA than the 2D cells. 

### 2.4. Protein Regions

**Figure 8:**
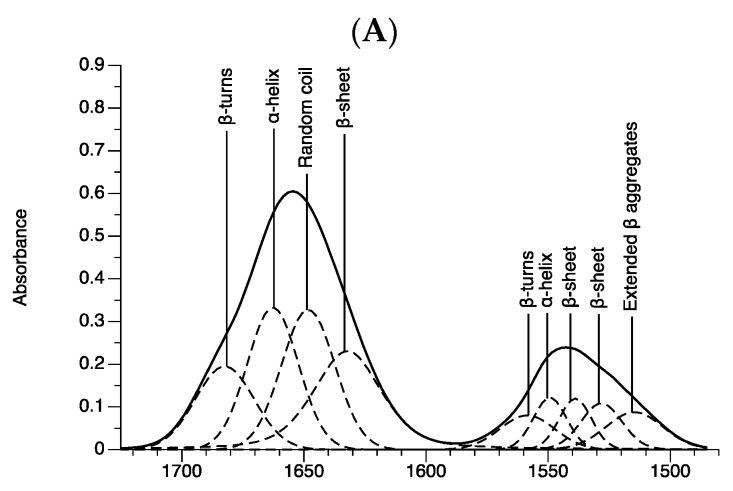

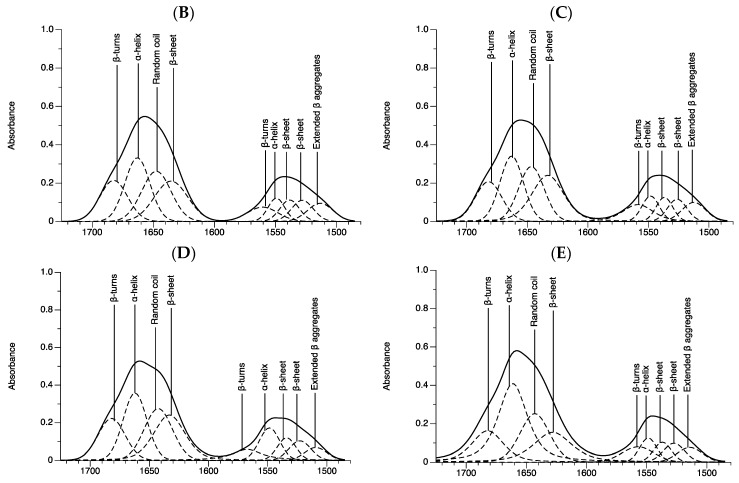
Average absorbance of amide I and II bands contour with best-fit 50% Lorentzian/Gaussian individual component bands for (**A**) 2D cells (RMS error is 0.003), (**B**) 5000 (RMS error = 0.004), (**C**) 8000 (RMS error = 0.004), (**D**) 10,000 (RMS error = 0.006), and (**E**) 20,000 (RMS error = 0.004) spheroid cells.

Based on the previous results, we found that amide I has potential for discriminating and characterizing the 2D cells and 3D spheroid cells and distinguishing between the 3D spheroid cells. The protein secondary structure was elucidated by curve fitting analysis. Normally, amide I (1600–1700 cm^−1^) is obtained from the stretching vibrations (*v*s) of C=O and C–N in the peptide backbone and is sensitive to a protein secondary structure change in the cell component [23,24]. The results show that the amide I region was divided into four sub-structures, including (1) β-pleated sheets at 1628–1635 cm^−1^, (2) random coil structure at 1641–1649 cm^−1^, (3) α-helix at 1660–1664 cm^−1^, and (4) β-turns structures at 1679–1683 cm^−1^ (Figure 8 and Table 2). In addition, the amide II region (1480–1600 cm^−1^) was elucidated to confirm the curve fitting analysis from amide I (Figure 8 and Table 2). The amide II was divided into five sub-structures, including (1) extended β aggregates (1508–1516 cm^−1^), (2) β-sheet (1522–1530 cm^−1^), (3) β-sheet (1534–1540 cm^−1^), (4) α-helix (1548–1550 cm^−1^), and (5) β-turns (1555–1567 cm^−1^). Those peak positions of the secondary structure of protein were assigned at similar regions to those previously reported [22,23,24,25,26,27]. 

Table 2 shows the peak positions and integral area of protein structure between the adherent (2D) and spheroid (3D) cells. We found that the peak positions of all protein secondary structures (β-sheets (1528, 1548, and 1632 cm^−1^), α-helix (1660 and 1548 cm^−1^), β-turns (1560 cm^−1^), random coil (1645 cm^−1^), and extended β aggregates (1513 cm^−1^) were significantly different between groups (Figure 8 and Table 2). The variation of peak position comes from the hydrogen bond interaction with the protein structure [23]. The peak positions were correlated to the protein secondary structure and protein function [25]. The differences in the protein secondary structure between 2D cells and 3D spheroid cells suggest that the two culture systems have different functional proteins. The integrated area of the peak position reveals the amount of the protein secondary structure [26]. A significantly higher integrated area of the α-helix (1548 and 1660 cm^−1^) and a lower random coil structure (1645 cm^−1^) were found in the 2D cells compared to the 3D spheroid cells (Table 2). Only the integrated area of the β-sheet in amide I (1631 cm^−1^)—and not in amide II (1526 and 1537 cm^−1^)—was significantly different among the 3D spheroid cells. The integrated area of the β-turns in the amide I area (1682 cm^−1^) was not significantly different between the 2D cells and 3D spheroid cells. Significant differences in the integrated area of β-turns in the amide II area (1560 cm^−1^) were only observed between the 2D and spheroids with 20,000 cells. β aggregates in amide II (1513 cm^−1^) were significantly different between 10,000 and 20,000 spheroid cells, and both 3D cells were significantly different from the other cell groups. The data suggest that the content of the α-helix in amide I and II (1548 and 1660 cm^−1^), random coil (1645 cm^−1^), and β-sheet (1631 cm^−1^) in amide I, and β-turns in amide II (1560 cm^−1^) structures could be used as markers in the protein regions for discriminating between the 2D cells and 3D spheroid cells and for monitoring spheroid cell growth.

## 3. Discussion

Melanoma is a heterogenic disease, for which single therapy is not enough to suppress melanoma cell growth. Combination targeted therapy (BRAF and MEK inhibitors) and immunotherapy (PD-1 blockade) were introduced to increase the survival rate [28]. Understanding the characteristics of melanoma could increase the successful treatment of melanoma with less adverse effects, particularly of secondary cancers [2]. Melanoma disease modeling was once entirely based on a 2D cell culture system, until a 3D melanoma culture system was developed [3]. The 3D melanoma culture system was introduced in three different ways, including (i) multicellular melanoma spheroids, multicellular melanoma spheroids in skin equivalent model [29], and skin or melanoma on chip [30]. Compared with the 2D cell culture system, all the 3D cell culture systems have several advantages, including: (i) representing in vivo cell morphology/structure, (ii) better representing cell-to-cell interactions and cell-to-extracellular matrix interactions, (iii) mimicking gene and protein expression, and (iv) mimicking tumor cell population, leading to better prediction of test compounds [5]. Most importantly, 3D cell culture systems facilitate precision medicine [31]. These advantages make 3D cell culture systems better suited to clinical management of the disease than a 2D cell culture system.

Multicellular tumor spheroids represent one type of 3D structure that is generated in vitro using cell culture systems. In their various forms, these may also be referred to as microtissues, embryoid bodies, organoids, or tumor spheres, and typically are spherical aggregates of proliferating, quiescent, and necrotic cells that in culture retain 3D architecture and tissue-specific functions [32]. In recent years, 3D spheroid culture systems have come to the fore in cancer research, drug discovery, and toxicology [32]. Interest in spheroids has grown because of the similarity to the way tumors develop, so they have relevance to the study of tumor biology, spheroids are amenable to generation in large numbers relatively consistent in size and shape, and spheroids can be counted, morphologically measured, and analyzed by a variety of techniques and instruments [32]. 

The most successful technique is ultra-low attachment [33], which was selected to create the 3D spheroid culture system in the current study. Typically, a variety of surface coatings are used to generate the ultra-low cell attachment: cell immobilization is inhibited, and cells are forced into a suspended state so that they clump together enabling 3D spheroid formation [5,32]. To generate 3D spheroids, ultra-low attachment (ULA) plates are coated with hydrophilic hydrogel. 

Despite it being increasingly evident that 3D cell culture technologies represent a better in vitro model than the 2D monolayer cell culture system, 3D cell culture systems have limitations, including: (i) size variation between batches, (ii) assay optimization to determine cellular response, and (iii) extraction of cells for single cell analysis [34]. The size variation of 3D spheroids can occur during spheroid formation between batches and can cause variation in spheroid investigations [4]. In the current study, we created variation between size of spheroids and we successfully used FTIR microspectroscopy to monitor changes caused by this variation at a molecular level. The variation of initial cell density mainly affected the amount of biocomponents, size variation, and cell death of spheroids [6]. FTIR microspectroscopy can thus be used to monitor the variation between spheroid groups by measuring the amounts of acryl chain of lipids and phosphodiester nucleic acids (DNA content) in the spheroid cells. The amount of lipid bilayer can be increased during apoptotic and necrotic cell death [26]. DNA content is more closely associated with necrotic cell death than apoptotic cell death [26]. In our study, a strong positive correlation was observed between lipid, DNA, and RNA content and induction of necrosis (propidium iodide) (Supplementary Table S1). This observation suggests that FTIR spectra gives a similar result as fluorescence staining. These regions should thus be used as a key factor for monitoring cell death induction during spheroid production and optimization. 

PCA analysis showed cell clustering in a group of cells between 5000, 8000, and 10,000 spheroid cells. A clear separation of cell clustering from PCA and cluster analysis was found at 20,000 spheroid cells. The number of necrotic cells dramatically increased when there were 20,000 spheroid cells. In previous studies, melanoma spheroids were commonly created using 5000 spheroid cells [14,15,35], which was a 3D spheroid used for common drug tests. Compared to the 2D cell culture system [36], the 3D spheroid culture system requires less cell seeding density. Based on our study, the increased cell seeding number might increase necrotic cell death at the core of the spheroid. Spheroid size and necrosis area varied between cell lines and cell numbers [37]. A 3D breast cancer cell culture was created using 50,000 spheroid cells, but no necrosis was reported [37]. In the present study, the cell density for creating a melanoma spheroid was between 5000 and 10,000 cells per spheroid, in order to prevent the occurrence of necrotic cell death in the spheroid, especially when conducting drug molecule efficacy testing. 

FTIR microspectroscopy can distinguish between 2D and 3D melanoma cell culture systems. Those differences can be discerned from the amount of alkyl chains of lipids, proteins (β-sheet, α-helix, and random coil), and DNA and RNA components. An increasing band of α-helix in 3D spheroid cells was also found when the cell seeding number was increased. Lipids and nucleic acids have roles in the phospholipid bilayer and/or cholesterol and DNA components [23]. The respective amount of lipid bilayer and DNA components is associated with cell stage and cell viability [22]. The change between protein content and expression between the 2D and 3D spheroid culture systems has been observed in colon cancer [38] and other types of cancers [38,39,40]. The protein secondary structure changes are the hallmarks of protein dynamics and are often related to protein function [41]. The concentration changes of β-pleated sheets, β-turns, α-helix, and random coil provide evidence of different protein functions that will affect cellular responses between 2D and 3D spheroid culture systems. Hence, there was a strong likelihood of success since the same measurement was performed and it can be further translated to a benchtop FTIR microscope with a globar source. 

In order to translate the method to the globar light source FTIR, there should be some parameter adjustments to improve the diffraction limit on the spatial resolution [42,43,44], such as (1) size of aperture, (2) magnification and reflective index of objectives, and (3) solid immersion approaches. An optimum size of aperture should be selected when translated to globar source FTIR [44,45] because a smaller aperture size can increase the diffraction limit on spatial resolution but reduce the signal-to-noise ratio of the spectra and increase the time of measurement [42,43,44,45,46]. Generally, the aperture size of the synchrotron light source ranged from 5 × 5 to 20 × 20 μm^2^, and the globar light source ranged from 15 × 15 to 150 × 150 μm^2^ [47]. Previous studies presented the successful use of globar light source FTIR for discriminating individual cells with an aperture size ranging from 30 × 30 to 50 × 50 μm^2^ [27,43]. The study of 2D and 3D breast cancer cell discrimination reported using an aperture size ranging from 170 × 170 to 180 × 180 μm^2^ [8,48]; hence, the optimum aperture size used in globar light source FTIR for cell study could range between 30 × 30 to 180 × 180 μm^2^. A high magnification and reflective index of objectives should be used to improve the diffraction limit on the spatial resolution, and signal-to-noise ratio [49]. Solid immersion approaches should be used, such as a micro-attenuated total reflection (ATR) objective [44] or an infrared transparent hemisphere (i.e., CaF_2_ or ZnS) [42,50,51]. Previous studies showed that CaF_2_ and ZnS hemispheres could improve the spatial resolution with 1.4–2.2 times higher than the conventional globar light source FTIR microspectroscopy [42,51]. The FTIR microspectroscopy is a promising tool for 3D cell biology studies. With a relatively long wavelength of infrared light of a globar source FTIR, the spatial resolution is limited to 3–30 μm [42,46]. The diameter of mammalian cells was 10–50 μm [42,46]. In our study, the diameter of human melanoma SK-MEL-2 cells was 9.6 ± 0.5 μm (n = 30); thus, it was possible to use globar source FTIR to disclose the biocomponents of the 2D and 3D cells.

FTIR spectra provide detailed information on the protein secondary structure, amount of specific biocomponents, and wavenumber, all of which contribute to the alteration of biological composition in different cell culture systems. Our results are strongly correlated with spheroid variations and cell death induction. It should be noted that FTIR microspectroscopy is a relatively fast and low-cost method, requiring low sample manipulation compared to other biological techniques [8]. Coupled with multivariate analysis, FTIR can be used to detect specific biomolecular markers (i.e., DNA backbone, unsaturated lipid, secondary structure of protein) inside cells, as reflected in the variation of spheroid cells [52]. Alternatively, FTIR microspectroscopy has been used to monitor stem-like cell populations in human esophageal normal and cancer cells [53] and resistant tumor cells [54]. Taken together, the use of FTIR microspectroscopy coupled with multivariate analysis could be promoted for classification and quality control of melanoma spheroids. 

## 4. Materials and Methods 

### 4.1. Cell Culture Reagents

The reagents and culture media (including Dulbecco’s modified Eagle’s medium of high glucose (DMEM), 0.25% trypsin-EDTA, and penicillin and streptomycin) were purchased from GIBCO^®^, Invitrogen (Grand Island, NY, USA). Fetal bovine serum (FBS) was purchased from GE Life Sciences (Parramatta, Australia).

### 4.2. Cell Line and Cell Culture

The human melanoma cell line (SK-MEL-2) (CLS-Cell lines Service, Eppelheim, Germany) was maintained in DMEM supplemented with 10% FBS, 100 units/mL of penicillin, and 100 µg/mL of streptomycin, at 37 °C in 5% CO_2_ atmosphere. The medium was changed every other day and the cells were cultured until they reached the exponential phase, when they were used in the experiments. For monolayer cells, the cells were seeded at 5 × 10^3^ cells/well in a 24-well plate. The monolayer cells were incubated for 5 days, then they were used for further analysis.

### 4.3. Spheroid Formation

The SK-MEL-2 cells were seeded at 5 × 10^3^, 8 × 10^3^, 10 × 10^3^, and 20 × 10^3^ cells/well on 1.5% w/v agarose in 96-well plates. The spheroids formed in approximately 5 days. The spheroids were inspected by a phase contrast inverted microscope (EVOS^TM^ FL auto 2, Thermo Fisher Scientific, Rockford, IL, USA) and their spheroid area and volume were measured. The spheroid volume was calculated by first determining the spheroid area and radius, then calculating volume from 4/3 × π × (radius)^3^.

### 4.4. Annexin V and Propidium Iodide Staining

The spheroids were plated into a 96-well fluorescence plate (Thermo Fisher Scientific, Rockford, IL, USA) then washed twice with staining buffer. Annexin V and propidium iodide (PI) staining (Biolegend, San Diego, CA, USA) were added as per the manufacturer’s instructions and incubated at 25 °C for 15 min. The fluorescence signals were measured using a fluorescence inverted microscope (EVOS^TM^ FL auto 2, Thermo Fisher Scientific, Rockford, IL, USA). The respective green and red color represented Annexin V and PI fluorescence signals. The quantitative fluorescence signals from Annexin V and propidium iodide (PI) were presented as a corrected total cellular fluorescence (CTCF) ratio. The CTCF ratio was calculated from Equation (1) [55].

CTCF = D_int_ − (A × F) (1)

D_int_ = integrated density of the spheroid
A = spheroid area
F = mean fluorescence of background readings

### 4.5. FTIR Data Acquisition

The FTIR samples were prepared as previously reported [56] with some modifications. The 2D and 3D SK-MEL-2 cells were trypsinized (final concentration of 0.25%) and centrifuged at 540 *g* for 5 min. These cell pellets were twice washed using 0.9% of NaCl (w/v) and re-suspended in 50 µL of 0.9% of NaCl (w/v). This step was gently performed to avoid the abrupt change of the osmolality between the culture media and the physiological saline solution. The drop of re-suspended cells was transferred onto a barium fluoride window (BaF_2_) and vacuum-dried for 30 min in a desiccator [17,56]. The cells on the window were rinsed with distilled water then vacuum-dried. This step was repeated to completely remove the salt. The washed and dried cell monolayer was kept in a desiccator prior to use. The spotted cells on the BaF_2_ window were analyzed in the transmission mode by a FTIR spectrometer (Bruker Vertex 70) connected to a Bruker Hyperion 2000 microscope (Bruker optic Inc, Ettlingen, Germany), using synchrotron radiation as the light source (Synchrotron Light Research Institute, Thailand) [57,58]. The FTIR spectrometer was coupled with a potassium bromide beam splitter and a MCT (HgCdTe) detector under liquid nitrogen. The scanning range was between 800 and 4000 cm^−1^ at a spectral resolution of 6 cm^−1^. Each cell drop was scanned 30–40 times to obtain 30–40 spectra. For each spectrum, 64 scans, 10 × 10 µm^2^ aperture size, were acquired using OPUS 6.5 software (Bruker optic Inc, Ettlingen, Germany). Using OPUS 6.5 software, we determined the water-compensation—spectra cut between 900 and 3000 cm^−1^—baseline correction and integration areas: between 2813 and 2992 cm^−1^ for the lipid region, 1480 and 1700 cm^−1^ for the amide I and II region, 1180 and 1280 cm^−1^ for the DNA region, 1040 and 1140 cm^−1^ for the RNA region, and 2813 and 2992 cm^−1^, with 937 and 1772 cm^−1^ for the whole cell region. After water compensation and baseline correction, the spectra with a peak height at the amide I region (1660 cm^−1^) lower than 0.4 a.u. was discarded, such that 20–26 spectra were obtained for each cell drop. There were three replications (3 drops from 3 spheroids) from each cell seeding condition (cell group), so 60–76 spectra were obtained per cell group for further analysis.

Multivariate analysis was performed using Unscrambler^®^ X software (version 10.2, CAMO Software AS, Oslo, Norway). Unsupervised methods were completed using principal component analysis (PCA) and hierarchical cluster analysis using Ward’s algorithm. The primary spectra were smoothed using the Savitzky–Golay method (polynomial order 3 with 13 smoothing points). Then, the spectra (between 2813 and 2992 cm^−1^, 937 and 1772 cm^−1^) were baseline corrected and normalized using Extended Multiplicative Signal Correction (EMSC), to correct for differences in sample thickness and light scattering artifacts before performing the multivariate analysis. The top two principal components (PCs) were chosen for analysis. Score plots (2D) and loading plots were used to display the clustering of the spectra and variations (wavenumber) from each range of spectra in the dataset. 

Unsupervised hierarchical cluster analysis of the FTIR spectral data sets was performed using Ward’s algorithm, utilizing a matrix defining the inter-spectral distances to identify and confirm the discrimination of the FTIR spectra from the PCA analysis. The spectral regions used in the cluster analysis were obtained from the loading plot, which mainly represents the biological components inside the cells. RStudio version 1.2.1355 was used to perform the hierarchical cluster analysis. 

Curve fitting was used to analyze the average absorbance spectra of the amide I and amide II regions (1480–1720 cm^−1^) so as to differentiate the secondary structure of the protein. A second derivative (Savitzky–Golay method with 13 points of smoothing) was used to estimate the protein secondary structure. The primary spectra were baseline corrected and deconvoluted into the specific areas for the amide I (1593–1723 cm^−1^) and amide II (1483–1590 cm^−1^) regions. The 50% Gaussian and Lorentzian function of the OPUS 6.5 software was used to perform the curve fitting. The peak position, integral area, and width were obtained from the curve fitting. The goodness of fit was determined by assessing the residual RMS error. The secondary structures of protein were assigned according to the previous reports [22,24,25,26,59].

### 4.6. Statistical Analysis

Spheroid volume, annexin V, and propidium iodide fluorescent intensity were expressed as means (±SD). The integration ratio, the secondary peak position, and integration area were expressed as means (±SD). The difference between groups was analyzed using a nonparametric Kruskal–Wallis test. The correlation between biological contents, fluorescence staining, and spheroid volume were analyzed using the Pearson’s correlation test. Statistical analyses were performed using SPSS 24.0 (SPSS Inc, IL, USA) and *p*-values below 0.05 were considered statistically significant. A graphic illustration was created using Datagraph version 4.3 (Visual data tools Inc, Chapel Hill, NC, USA).

## 5. Conclusions

FTIR microspectroscopy coupled with the multivariate analysis could be used to discriminate and monitor the changes that occur between 2D and 3D-spheroid melanoma cell growth. Significant changes were observed in protein secondary structures, and the phospholipid and DNA contents between the two culture systems. The FTIR technique successfully monitored variation between spheroid size and cell number at the molecular level. The amount of phospholipid and DNA principally accounted for variations tracked between the size and viability of the spheroids. FTIR microspectroscopy is thus an alternative tool for monitoring the process of spheroid production and optimization. This finding could be used to enhance the transitioning process from 2D cell culture to 3D spheroid culture vis-à-vis melanoma disease modeling.

## Figures and Tables

**Table 1:** Five-day-old SK-MEL-2 spheroid volume and cell death fluorescence intensity. Different letters in the same column indicate significant differences between samples (*p* < 0.05).

Initial Cell Numbers	Volume (mm^3^) (*n* = 3)	Annexin V Intensity (× 10^7^ CTCF ^1^) (*n* = 2)	Propidium Iodide Intensity (× 10^7^ CTCF) (*n* = 2)
5000	40.3 ± 5.4 ^a^	1.0 ± 0.2 ^a^	0.7 ± 0.0 ^a^
8000	68.8 ± 2.7 ^b^	0.8 ± 0.1 ^a^	1.5 ± 0.4 ^a^
10,000	86.3 ± 1.7 ^c^	2.5 ± 0.0 ^a^	1.4 ± 0.1 ^a^
20,000	97.7 ± 2.0 ^d^	2.3 ± 0.4 ^a^	4.0 ± 0.5 ^a^

^1^ CTCF is corrected total cell fluorescence.

**Table 2:** Peak positions and integral area of secondary structure of protein between the 2D cells and groups of 3D spheroid cells. Data are expressed as mean ± standard deviation (SD) of three replicates. Different superscripts in the same column indicate significant differences between groups (*p* < 0.05).

Group	Amide I				Amide II				
	β-Turns	α-Helix	Random Coil	β-Sheet	β-Turns	α-Helix	β-Sheet	β-Sheet	Extended β Aggregates
	**Peak position (cm^−1^)**								
**2D**	1681.5 ± 1.8	1662.0 ± 1.3 ^a,b^	1647.4 ± 1.1 ^b,c^	1631.8 ± 0.3 ^a,b^	1560.1 ± 2.6 ^a,b,c^	1549.7 ± 1.1 ^b^	1539.2 ± 1.2 ^b^	1528.6 ± 1.7 ^b^	1514.7 ± 1.2 ^b^
**5000**	1682.4 ± 0.2	1663.4 ± 0.6 ^b^	1648.4 ± 1.0 ^c^	1634.6 ± 0.8 ^b^	1560.1 ± 0.7 ^b,c^	1549.1 ± 0.3 ^a,b^	1538.0 ± 0.3 ^a,b^	1527.4 ± 0.5 ^a,b^	1513.1 ± 0.2 ^a,b^
**8000**	1681.9 ± 0.1	1662.7 ± 0.2 ^a,b^	1646.5 ± 0.1 ^b,c^	1633.1 ± 0.1 ^a,b^	1558.1 ± 0.5 ^a,b^	1548.1 ± 0.1 ^a,b^	1537.1 ± 0.3 ^a^	1526.7 ± 0.4 ^a,b^	1512.9 ± 0.3 ^a,b^
**10,000**	1682.6 ± 0.8	1662.2 ± 0.1 ^a^	1642.1 ± 0.4 ^a^	1631.6 ± 1.9 ^a,b^	1566.4 ± 0.8 ^c^	1549.4 ± 1.0 ^a,b^	1535.5 ± 1.8 ^a^	1524.0 ± 1.8 ^a^	1509.2 ± 0.9 ^a^
**20,000**	1681.8 ± 0.3	1660.9 ± 0.3 ^a^	1643.1 ± 0.6 ^b^	1627.6 ± 1.1 ^a^	1556.2 ± 0.9 ^a^	1548.3 ± 0.3 ^b^	1536.3 ± 0.6 ^a^	1526.3 ± 1.2 ^a,b^	1513.3 ± 1.1 ^a,b^
	**Integral area (%)**								
**2D**	13.2 ± 2.0	20.4 ± 0.4 ^a^	20.7 ± 0.3 ^b^	13.7 ± 1.8 ^a,b^	4.7 ± 0.6 ^a,b^	7.7 ± 0.1 ^a^	7.3 ± 0.2	6.8 ± 0.1	5.5 ± 0.3 ^c^
**5000**	13.3 ± 0.7	21.0 ± 0.8 ^a,b^	17.7 ± 0.5 ^a,b^	14.7 ± 1.0 ^b^	5.0 ± 0.1 ^a,b^	7.9 ± 0.2 ^a^	7.4 ± 0.1	7.1 ± 0.1	5.9 ± 0.3 ^c^
**8000**	13.0 ± 0.3	21.3 ± 0.4 ^a,b^	17.3 ± 0.2 ^a^	14.3 ± 0.3 ^b^	5.3 ± 0.1 ^b^	7.9 ± 0.2 ^a,b^	7.5 ± 0.1	7.1 ± 0.0	6.2 ± 0.1 ^c^
**10,000**	14.1 ± 0.7	25.1 ± 1.9 ^b,c^	18.4 ± 0.6 ^a,b^	9.7 ± 1.0 ^a^	4.1 ± 0.3 ^a^	9.9 ± 1.4 ^b^	7.5 ± 0.2	7.3 ± 0.5	3.9 ± 0.7 ^a^
**20,000**	11.6 ± 0.4	27.1 ± 0.9 ^c^	18.0 ± 0.8 ^a,b^	10.6 ± 1.3 ^a,b^	5.5 ± 0.1 ^c^	8.5 ± 0.2 ^a,b^	7.2 ± 0.2	6.6 ± 0.2	4.9 ± 0.4 ^b^

## Supplementary Materials

The following are available online at https://www.mdpi.com/1422-0067/21/11/4141/s1. Table S1: Pearson correlation coefficient between biocomponent of spheroid cells and mode of cell death and spheroid volume.
