# Supplementary material for: Evaluation of Melanoma (SK-MEL-2) Cell Growth between Three-Dimensional (3D) and Two-Dimensional (2D) Cell Cultures with Fourier Transform Infrared (FTIR) Microspectroscopy
Source: Int J Mol Sci. 2020 Jun 10;21(11):4141. doi: 10.3390/ijms21114141 (PMC7312007; doi:10.3390/ijms21114141)
Supplement: Supplementary file 1 [file ijms-21-04141-s001.pdf]

International Journal of Molecular Sciences

MDPI

Supplementary Materials

Supplementary Table S1. Pearson correlation coefficient between the biocomponent of spheroid cells and mode of cell death and spheroid volume.

	Annexin V	Propidium Iodide	Lipid	Amide I	Amide II	DNA	RNA	Volume
**Annexin V**	1.000	0.544	0.533	-0.542	-0.595*	0.542	0.224	0.774**
		p=0.067	p=0.074	p=0.069	p=0.041	p=0.068	p=0.484	p=0.003
**Propidium iodide**	0.544	1.000	0.987**	-0.997**	-0.992**	0.928**	0.912**	0.780**
	p=0.067		p<0.001	p<0.001	p<0.001	p<0.001	p<0.001	p=0.003
**Lipid**	0.533	0.987**	1.000	-0.978**	-0.980**	0.895**	0.877**	0.697**
	p=0.074	p<0.001		p<0.001	p<0.001	p<0.001	p<0.001	p<0.012
**Amide I**	-0.542	-0.997**	-0.978**	1.000	0.990**	-0.914**	-0.903**	-0.800**
	p=0.069	p<0.001	p<0.001		p<0.001	p<0.001	p<0.001	p=0.002
**Amide II**	-0.595**	-0.992*	-0.980**	0.990**	1.000	-0.933**	-0.875**	-0.798**
	p=0.041	p<0.001	p<0.001	p<0.001		p<0.001	p<0.001	p=0.002
**DNA**	0.542	0.928**	0.895**	-0.914**	-0.993**	1.000	0.869**	0.763**
	p=0.068	p<0.001	p<0.001	p<0.001	p<0.001		p<0.001	p=0.004
**RNA**	0.224	0.912**	0.877**	-0.903**	-0.875**	0.869**	1.00	0.619**
	p=0.484	p<0.001	p<0.001	p<0.001	p<0.001	p<0.001		p=0.032
**Volume**	0.774**	0.780**	0.697**	-0.800**	-0.798**	0.763**	0.619**	1.000
	p=0.003	p=0.003	p<0.012	p=0.002	p=0.002	p=0.004	p=0.032	

Pearson correlation (r) near to +1 or −1 indicates a strong relationship, and near 0 indicates a weak or no relationship. *, ** Correlation is significant at p <0.05 and p <0.01, respectively.
